# Molecular Characterization and Phylogenetic Analysis of a Variant Recombinant Porcine Epidemic Diarrhea Virus Strain in China

**DOI:** 10.3390/ani12172189

**Published:** 2022-08-25

**Authors:** Xiaoliang Hu, Yuexiao Lian, Yucan He, Xiangxiao Liu, Zhige Tian, Yi Dai, Mengyuan Liu, Huayan Fan, Yue Shi, Feng Cong

**Affiliations:** 1 Faculty of Agriculture, Forestry and Food Engineering, Yibin Key Laboratory of Zoological Diversity and Ecological Conservation, Yibin Animal and Plant Inspection and Quarantine Engineering Technology Research Center, Yibin University, Yibin 644000, China; 2 Guangdong Laboratory Animals Monitoring Institute, Guangdong Provincial Key Laboratory of Laboratory Animals, Guangzhou 510633, China; 3 Beijing Senkang Biotech Development Co., Ltd., Beijing 100000, China

**Keywords:** porcine epidemic diarrhea virus, variant, insertion, pathogenicity, E gene

## Abstract

**Simple Summary:**

We successfully isolated and identified PEDV strain SC-YB73. The sequence analysis of the SC-YB73 genome identified a six-nucleotide insertion in the E gene, which has not previously been detected in PEDV strains. The phylogenetic analysis based on the complete genome showed that SC-YB73 was clustered in variant subgroup GII-a, which is widely prevalent in the Chinese pig population. The recombination analysis suggested that SC-YB73 originated from the recombination of GDS47, US PEDV prototype-like strains TW/Yunlin550/2018, and COL/Cundinamarca/2014. In future research, we aim to evaluate the function of E-gene insertions using in vitro cellular culture and in vivo animal experiments.

**Abstract:**

Since 2010, a variant of porcine epidemic diarrhea virus (PEDV) has re-emerged in several provinces of China, resulting in severe economic losses for the pork industry. Here, we isolated and identified a variant PEDV strain, SC-YB73, in Guangdong Province, China. The pathological observations of jejunum showed atrophy of villi and edema in the lamina propria. The sequence analysis of the viral genome identified a six-nucleotide insertion in the E gene, which has not previously been detected in PEDV strains. Furthermore, 50 nucleotide sites were unique in SC-YB73 compared with 27 other PEDV strains. The phylogenetic analysis based on the complete genome showed that SC-YB73 was clustered in variant subgroup GII-a, which is widely prevalent in the Chinese pig population. The recombination analysis suggested that SC-YB73 originated from the recombination of GDS47, US PEDV prototype-like strains TW/Yunlin550/2018, and COL/Cundinamarca/2014. In the present study, we isolated and genetically characterized a variant PEDV strain, thus providing essential information for the control of PED outbreaks in China.

## 1. Introduction

Porcine epidemic diarrhea virus (PEDV) is the etiological agent of porcine epidemic diarrhea (PED). First identified in 1978, the virus can cause severe diarrhea with high morbidity in neonatal piglets [1]. PEDV is an enveloped, single-stranded, positive-sense RNA virus belonging to genus *Alphacoronavirus* and possessing a large (28 kb) genome. Two-thirds of the RNA genome is comprised of open reading frames (ORFs) 1a and 1b, encoding RNA replicase, while the 3′ one-third of the genome is comprised of genes encoding structural and non-structural proteins [2,3,4,5].

From 1984 to 2010, no large-scale PED outbreaks were recorded in China, with only sporadic occurrences in the pig population [6]. At the end of 2010, however, an outbreak involving variant PEDV strains rapidly spread across southern China, resulting in considerable economic losses for the pig industry [7,8]. Based on their complete genome sequences, PEDV can be classified into genotype I and II (GI and GII) groups [9,10]. The GI group contains classical strains and includes the GI-a, GI-b, and GI-c subgroups. The GII group comprises so-called variant strains and includes the GII-a and GII-b subgroups. In China, GII group variants are highly virulent and dominant pandemic strains in the pig population [10,11,12], whereas classical strains in the GI group, including GI-a, GI-b, and GI-c, are uncommon [13,14,15].

In this study, we isolated PEDV strain SC-YB73 from the intestinal contents of piglets in Guangdong Province, China. To better understand the molecular characteristics of this isolate, we obtained its complete genome sequence. This study provides molecular and phylogenetic information on a Chinese isolate of PEDV, which may help elucidate the genetic evolution of PEDV in China.

## 2. Methods

### 2.1. Ethics Statement

Fecal and small-intestine tissues were collected by a farmer and given to us for pathogen diagnosis. In this process, we did not come into direct contact with the animal samples. 

### 2.2. Viral Isolation and Identification

The study site was a sow farm in Guangdong, China. The farm contained 200 pigs, which were not immunized with any PEDV vaccine prior to conception. In 2019, 20 out of 100 piglets on the farm developed yellow watery diarrhea, vomiting, and rapid weight loss, with death occurring within two days. Two samples were obtained from each dead piglet, and two samples were collected from dead piglets without apparent clinical signs. Previously established polymerase chain reaction (PCR) protocols were performed to detect four major diarrhea-associated viruses, i.e., PEDV [16], porcine deltacoronavirus (PDCoV) [17], transmissible gastroenteritis virus (TGEV) [16], and porcine rotavirus (PoRV) [18].

Small-intestine tissues were suspended in 20% (*w*/*v*) Dulbecco’s modified Eagle medium (DMEM; Gibco, Grand Island, NY, USA); then, they were vortexed and centrifuged at 5000× *g* for 5 min to harvest the supernatant. The supernatant was filtered through a 0.22-μm filter (Millipore, Billerica, MA, USA) and inoculated with the VERO-E6 cell line (ATCC; CCL-81). After three rounds of purification using a plaque assay [19], the virus was purified and harvested through one cycle of freezing and thawing. Virus titer was measured using the Reed–Muench method [20].

### 2.3. Extraction of Viral RNA, Reverse Transcription PCR (RT-PCR), and Complete Genome Sequencing

Viral RNA extraction and RT-PCR analysis were performed as previously described [16]. The 15 pairs of primers used are listed in Table 1. Two primers (5′ RACE and 3′ RACE) were employed to confirm the 5′ and 3′ ends of the viral genome via rapid amplification of cDNA ends (RACE) using a RACE cDNA Amplification kit (Invitrogen, Carlsbad, CA, USA) (Table 1). The PCR products were run on agarose gels, and correctly sized amplicons were observed. The PCR products were then purified using an Axygen Gel Extraction kit (Axygen, Union City, CA, USA) and cloned into the pMD18-T vector (TaKaRa, Tokyo, Japan). Three to five independent clones of each PEDV amplicon were sequenced. DNA was sequenced using an ABI 3730XL Sanger-based genetic analyzer (Applied Biosystems, Waltham, MA, USA).

### 2.4. Electron Microscopy and Pathology

Electron microscopy was performed following previous research [16]. Hematoxylin and eosin (H&E) staining of ileum samples was carried out according to the protocols described in [21].

### 2.5. Sequence Alignment and Phylogenetic and Recombination Analyses

Phylogenetic trees based on the complete genome and spike sequences were constructed using the neighbor joining (NJ) method in MEGA v4.0. Bootstrap values were estimated for 1000 replicates. Simplot v3.5.1 was used for nucleotide sequence comparison of SC-YB73 to the reference PEDV strains. The sequences obtained in this study were assembled and submitted to GenBank (accession number MT263014).

## 3. Results

### 3.1. Virus Isolation and Identification

**Figure 1:**
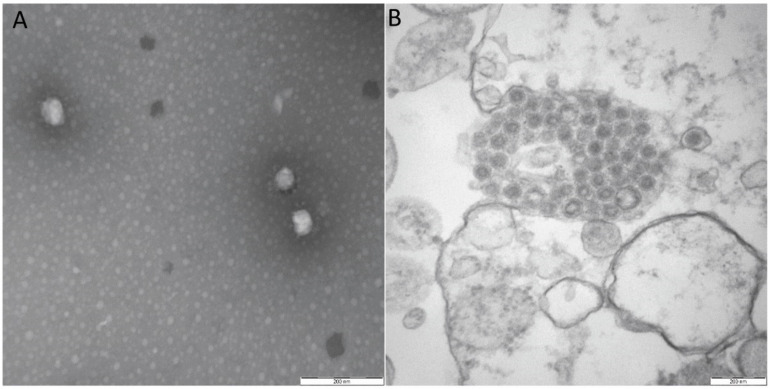
(**A**) Electron micrograph of purified isolate negatively stained with 2% phosphotungstic acid. (**B**) Ultra-thin sections of infected VERO-E6 cells displaying typical viral particles, organized as paracrystalline structures within cytosol. Scale bar, 200 nm.

**Figure 2:**
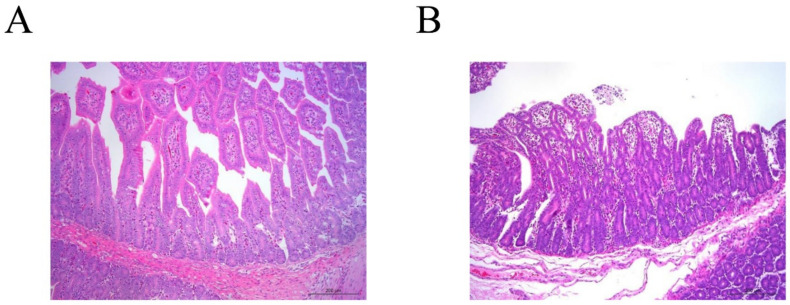
Pathology of jejunum villi of piglet samples: (**A**) control and (**B**) diseased piglets.

The PCR results confirmed that the two small-intestine samples were only positive for the PEDV strain SC-YB73 (data not shown). The other two samples were negative for PEDV, PDCoV, TGEV, and PoRV. At the beginning of passage five (P5), typical cytopathic effects (CPE) were found in VERO-E6 cells (data not shown). Following this, the SC-YB73 strain was purified through three rounds of plaque cloning and serially passaged for three generations in VERO-E6 cells. SC-YB73 titer (6.5 × 10^6^ TCID_50_/mL) was measured in VERO-E6 cells. The classical coronavirus shape was confirmed using electron microscopy, with virions measuring approximately 150 nm in diameter (Figure 1A,B). The histopathological analysis showed the shedding of intestinal villi, the degeneration and necrosis of mucosal epithelium, the infiltration of inflammatory cells in the mucosa and submucosal interstitium, the edema of submucosa and muscularis, and the congestion of blood vessels (Figure 2A,B).

### 3.2. Phylogenetic Analysis and Recombination of SC-YB73

**Figure 3:**
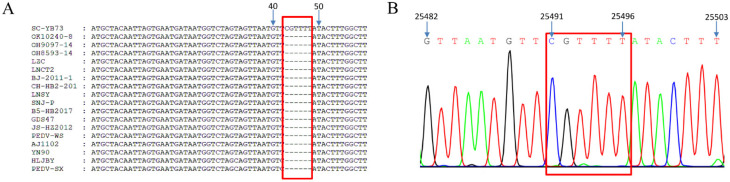
Multiple sequence alignment of E gene of PEDV strains (**A**) and partial-Sanger-sequencing peak map for SC-YB73 (**B**). The red box was present the six nucleotide acid insertions.

**Figure 4:**
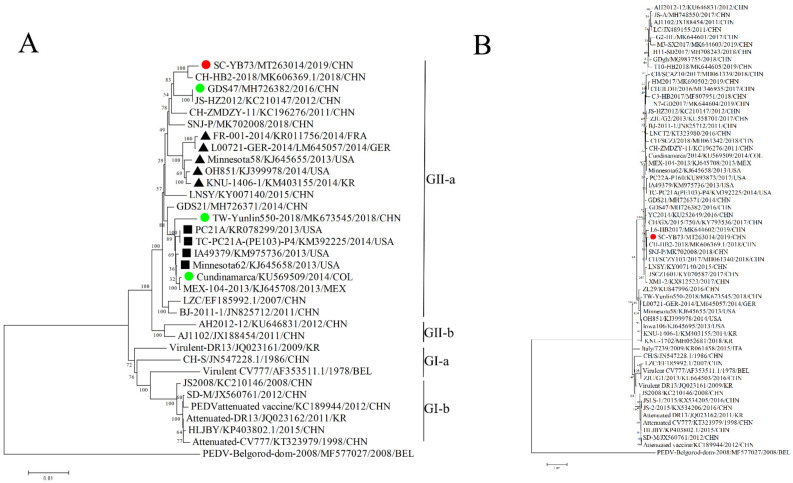
Phylogenetic analyses of complete sequence (**A**) and spike sequence (**B**) regions of SC-YB73 and most closely related strains in GenBank for which whole genome sequences were available. Neighbor joining was used for the construction of the phylogenetic tree with 1000 bootstrap replicates shown at branches. Scale bar represents *p*-distance. Red circle represents isolated strain in this study. Green circles represent possible recombinant strains. Black triangles represent US-PEDV-S-INDEL-variant-like strains. Black squares represent US PEDV prototype-like strains. CHN, China; USA, United States of America; GER, Germany; BEL, Belgium; COL, Columbia; KR, Republic of Korea; MEX, Mexico; FRA, France; ITA, Italy.

**Figure 5:**
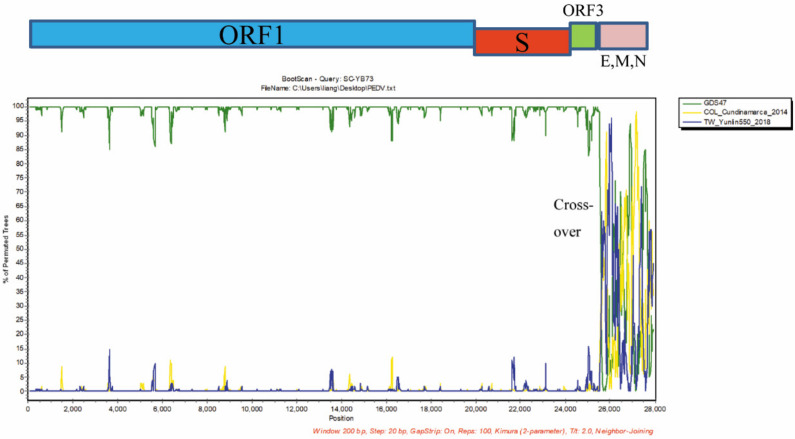
Recombination analysis of PEDV strains. Crossover region in SC-YB73 genome was detected with Simplot v3.5.1. Y-axis shows percentage of permuted trees employing a sliding window of 200 nucleotides (nt) and step size of 20 nt. Other parameters used included Kimura (2-parameter) distance model, 2.0 Ts/Tv ratio, neighbor-joining tree model, and 1000 bootstrap replicates. COL/Cundinamarca/2014:KU569509/2014/COL; GDS47:MH726382/2016/CHN; TW/Yunlin550/2018:MK673545/2019/CHN.

The genome sequence of the SC-YB73 strain was 28,040 nucleotide (nt) long. Whole-genome sequence alignment showed nucleotide homologies of 95.9% (CH/HB2/2018) to 99.6% (85/7/c40) between the SC-YB73 strain and 27 PEDV domestic reference strains. Based on the complete genome sequence, 50 unique positions were identified in SC-YB73 that were not found in the 27 other strains, suggesting that SC-YB73 possesses several novel characteristics (Table S1). A comparative sequence analysis identified a 6 nt insertion in the E gene of SC-YB73 not present in the other PEDV strains. The insertion did not change the existing ORF, with only two additional amino acids being produced (Figure 3A,B). The phylogenetic analysis indicated that the SC-YB73 strain, along with domestic strains CH-HB2-2018, GDS47, JS-HZ2012, CH_ZMDZY_11, and SNJ-P, belonged to the GII-a subgroup and formed a single branch. In addition, the US PEDV prototype-like strain GDS21 isolated in Guangdong in 2014 was closely related to PC21A, TC-PC21A-(PE103)-P4, IA49379, TW-Yulin550-2018, and COL/Cundinamarca/2014. These findings suggest that different PEDV strains, i.e., SC-YB73, GDS47, and GDS21, have been circulating in Guangdong since 2014 (Figure 4A). The S gene of SC-YB73 was closely clustered with domestic strains CH-HB2-2018 and SNJ-P (Figure 4B). A Simplot (v3.5.1) analysis was performed to identify possible recombination events and breakpoints in the complete nucleotide sequence of the SC-YB73 genome. The results indicated that SC-YB73 is probably a recombinant from GDS47, US PEDV prototype-like strains TW/Yunlin550/2018, and COL/Cundinamarca/2014, with crossover events having been detected (Figure 5) and recombination event breakpoints within the E, M, and N genes.

## 4. Discussion

As an evolutionary driving force, mutations such as point mutations, insertions, and deletions in structural and non-structural proteins have led to changes in the tropism and virulence of coronaviruses [22,23,24]. For example, deletions in the spike protein and ORF3 in TGEV result in a tropism shift from the intestinal to respiratory tract [25,26]. Deletions in ORF3abc in canine coronavirus HLJ-073 result in changes in cell tropism [24]. In the case of PEDV, deletions and insertions in the spike protein and ORF3 [27,28,29], as well as mutations in those genes, are closely related to viral replication and pathogenicity [2,4,5,30]. For example, HM2017 contains two insertions in the S gene [27]; HLJBY shows a 133-amino-acid deletion in ORF3 [28]; and 15 novel PEDV variants identified in Japan exhibit large genomic deletions [29]. Here, using PCR and pathological analyses, we isolated a novel PEDV strain, SC-YB73, in China. Based on sequencing analysis, no deletions were found in the complete sequence, and six nucleotides were inserted in the E gene without disrupting the ORF, resulting in two additional amino acids. This is the first report of an insertion in the E gene. Previous studies have suggested that the function of the E and M proteins in murine hepatitis virus (MHV) is to promote particle formation and secretion [31,32]. Whether the biological function of the SC-YB73 E protein affects pathogenicity needs further study. In addition, SC-YB73 may be virulent to piglets and exhibited 50 unique positions in the complete genome compared with 27 other strains. The positions in SC-YB73 were distinct from any other Chinese and non-Chinese strains, suggesting that the strain has antigenic variation or different biological activity and underwent a rapid evolutionary process. Further experiments are needed to evaluate the pathogenicity of SC-YB73 and the relationship between pathogenicity and the unique positions in the complete genome.

Another evolutionary driver is recombination, which plays an important role in the evolution of PEDV, leading to the emergence of highly virulent and immunogenic mutant strains (6,10). In this study, SC-YB73 showed evidence of potential recombination events from GDS47, US PEDV prototype strains TW/Yunlin550/2018, and COL/Cundinamarca/2014. Furthermore, the analyses confirmed the probable occurrence of recombination in the E, M, and N genes of SC-YB73. Thus, these results suggest that SC-YB73 was generated via the recombination of Guangdong circulating strains and US PEDV prototype strains. This represents a potential biosafety concern, as foreign strains can cause antigen mutations through recombination with circulating strains. In addition, recombination within subgroups is a common phenomenon and a driving force of PEDV evolution. Therefore, there is a growing need for novel effective and safe vaccines against PEDV. It is also important that PEDV outbreaks be examined in the context of new recombinant variants and that preventive measures against antigenic variation be considered.

## 5. Conclusions

We successfully isolated and identified PEDV strain SC-YB73. Based on a comparative sequence analysis with other PEDV strains, we gained a comprehensive understanding of the mutation and recombination of the PEDV strain. In future research, we aim to evaluate the function of E-gene insertions using in vitro cellular culture and in vivo animal experiments.

## Figures and Tables

**Table 1:** Primers used for identifying and sequencing the PEDV SC-YB73 strain.

Name	Sequence (5′-3′)	Position
Porf3-U	GGAGCTCAATGTAGTTCCAA	24,886–24,905
Porf3-L	AGCTGCTTTACCATTGAGGA	25,185–25,204
1-F	AGCTCTTTCTCTAGACTCTT	32–51
1-R	AGCTGCTCCCAAGCTGCGCT	1511–1530
2-F	TTTTTGAATGACTCGAGCAT	1331–1350
2-R	TAAACTGGGTCAATGGTTCT	3011–3030
3-F	GAATTAGAAGAGACGACATT	2831–2850
3-R	TGTCATAATTAGCATCACCA	5011–5030
4-F	TACAAATTCCAATTTGGATT	4831–4850
4-R	AATAAAAGTGCAGCCTGGAC	7011–7030
5-F	ATGTTTTCCTTGGCTGCGAT	6831–6850
5-R	TCAAAAGAGCCTACGAACTT	9011–9030
6-F	TTGTACTTTTTGTGCACTAA	8831–8850
6-R	GTTAGCAACCATATACTTAA	10,911–10,930
7-F	CTACGGTATTCTCTACTGGT	10,831–10,850
7-R	AGAACTTAACGCATTTAAGC	13,131–13,150
8-F	ACCGAGTATACTATGATGGA	12,931–12,950
8-R	GTTTTGTTGTGGCGGTAGTT	16,011–16,030
9-F	ACAGGTTGGCAAATGATGTC	15,731–15,750
9-R	CGGTATATTTACAGACATCC	19,011–19,030
10-F	GTTAGAGATGGTACTGTTGA	18,811–18,830
10-R	GGGCCTAATGTTTTAATGCT	21,021–21,040
11-F	CTGTGCTGGCCAACATCCAA	20,831–20,850
11-R	ATTAGAATGGTAGAAGAAAC	22,831–22,850
12-F	GCTTTAGAGGTGAGGGTATC	22,600–22,619
12-R	ATCACCCGGTACAAGTACTG	24,100–24,119
13-F	GTCAAATCGCAATCTCAGCG	24,000–24,019
13-R	TATAATAAGCAGGAAAAAGA	25,516–25,535
14-F	TCAATTCAACTAGACGAGTA	25,430–25,449
14-R	TCTGTTCTTGGACTGGTTAC	26,986–27,005
15-F	CTACTTCACGTGCAAACTCA	26,806–26,825
15-R	TATCAACACCGTCAGGTCTT	28,016–28,035
5′RACE	GCCCACATACGCACTAAGCT	501–520
3′RACE	ACTGGCTTATTCTGGCTATG	27,721–27,740

## Data Availability

The data presented in this study are openly available in GenBank database, accession number [MT263014].

## Supplementary Materials

The following supporting information can be downloaded at: https://www.mdpi.com/article/10.3390/ani12172189/s1, Table S1: Multiple sequence alignment of PEDV strains.

## Availability of Data and Materials

The DNA sequences obtained in this study were submitted to the GenBank database (accession number: MT263014).

## Abbreviations

Porcine epidemic diarrhea virus (PEDV); open reading frames (ORFs); porcine deltacoronavirus (PDCoV); transmissible gastroenteritis virus (TGEV); porcine rotavirus (PoRV); rapid amplification of cDNA ends (RACE); neighbor joining (NJ); nucleotides (nt); median tissue culture infective dose (TCID_50_); insertions and deletions (INDELs); Dulbecco’s modified Eagle medium (DMEM).
