# Supplementary material for: Molecular Characterization and Phylogenetic Analysis of a Variant Recombinant Porcine Epidemic Diarrhea Virus Strain in China
Source: Animals (Basel). 2022 Aug 25;12(17):2189. doi: 10.3390/ani12172189 (PMC9454955; doi:10.3390/ani12172189)
Supplement: Supplementary file 1 [file animals-12-02189-s001.zip › animals-1865519-supplementary.pdf]

Table S1 Multiple sequence alignment of PEDV strains.

Name	524	693	1518	1555	2280	3519	4784	6228	6438	6699	6777	7859	8298	8328	8375	9862	10569
SC-YB73/MT263014/2019/CHN	G	T	C	T	C	T	T	T	G	T	T	T	G	A	C	T	C
CH-HB2-2018/MK606369.1/2018/CHN	A	G	T	C	T	C	A	C	A	C	C	G	T	G	A	G	A
CH/JLDH/2016/MF346935/2017/CHN	A	G	T	C	T	C	A	C	A	C	C	G	T	G	A	G	A
CHN_SH-2016-4/2016/ MG837012/2016/CHN	A	G	T	C	T	C	A	C	A	C	C	G	T	G	A	G	A
LNSY/KY007140/2015/CHN	A	G	T	C	T	C	A	C	A	C	C	G	T	G	A	G	A
AH2012_12/KU646831/2012/CHN	A	G	T	C	T	C	A	C	A	C	C	G	T	G	A	G	A
85-7-C40/KY486714/2015/CHN	A	G	T	C	T	C	A	C	A	C	C	G	T	G	A	G	A
85-7 mutant/ KX839247/2015/CHN	A	G	T	C	T	C	A	C	A	C	C	G	T	G	A	G	A
CH/S/JN547228.1/1986/CHN	A	A	T	C	T	C	A	C	A	C	C	G	T	G	A	G	A
SNJ-P/MK702008/2018/CHN	A	A	T	C	T	C	A	C	A	C	C	G	T	G	A	G	A
OH9097-14/ KP641663/2014/USA	A	A	T	C	T	C	A	C	A	C	C	G	T	G	A	G	A
B5-HB2017/ MF807952/2017/CHN	A	A	T	C	T	C	A	C	A	C	C	G	T	G	A	G	A
LZC/EF185992.1/2007/CHN	A	G	T	C	T	C	A	C	A	C	C	G	T	G	A	G	A
HLJBY/KP403802.1/2015/CHN	A	G	T	C	T	C	A	C	A	C	C	G	T	G	A	G	A
CH_HNLH_2015/ KT199103/2015/CHN	A	G	T	C	T	C	A	C	A	C	C	G	T	G	A	G	A
BJ-2011-1/JN825712/2011/CHN	A	G	T	C	T	C	A	C	A	C	C	G	T	G	A	G	A
AJ1102/JX188454/2011/CHN	A	G	T	C	T	C	A	C	A	C	C	G	T	G	A	G	A
CH_FJND-3_2011/JQ282909/2011/CHN	A	G	T	C	T	C	A	C	A	C	C	G	T	G	A	G	A

CH/GDZHDM/1401/ KX016034/2014/CHN	A	G	T	C	T	C	A	C	A	C	C	G	T	G	A	G	A
CH/YNKM-8/2013/ KF761675/2013/CHN	A	G	T	C	T	C	A	C	A	C	C	G	T	G	A	G	A
CH/ZJCX-1/2012/KF840537/2012/CHN	A	G	T	C	T	C	A	C	A	C	C	G	T	G	A	G	A
GDS47/MH726382/2016/CHN	A	G	T	C	T	C	A	C	A	C	C	G	T	G	A	G	A
JS-HZ2012/KC210147/2012/CHN	A	G	T	C	T	C	A	C	A	C	C	G	T	G	A	G	A
LNCT2/KT323980/2016/CHN	A	G	T	C	T	C	A	C	A	C	C	G	T	G	A	G	A
PEDV-SX/ KY420075/2015/CHN	A	G	T	C	T	C	A	C	A	C	C	G	T	G	A	G	A
PEDV-WS/ KM609213/2014/CHN	A	G	T	C	T	C	A	C	A	C	C	G	T	G	A	G	A
YN90/ KT021231/2014/CHN	A	G	T	C	T	C	A	C	A	C	C	G	T	G	A	G	A
OK10240-8_2017	A	A	T	C	T	C	A	C	A	C	C	G	T	G	A	G	A

Table S1 Multiple sequence alignment of PEDV strains (continue).

Name	13515	14340	14361	14370	14391	14406	14419	14442	14492	15458	16572	16659	17311	17324	17334	18010	19661
SC-YB73/MT263014/2019/CHN	T	C	A	G	A	G	C	C	C	A	A	C	T	C	T	T	G
CH-HB2-2018/MK606369.1/2018/CHN	C	G	G	T	G	T	T	A	T	C	C	T	C	A	C	C	A
CH/JLDH/2016/MF346935/2017/CHN	C	G	G	T	G	T	T	A	T	C	C	T	C	A	C	C	A
CHN/SH-2016-4/2016/ MG837012/2016/CHN	C	G	G	T	G	T	T	A	T	C	C	T	C	A	C	C	A
LNSY/KY007140/2015/CHN	C	G	G	T	G	T	T	A	T	C	C	T	C	A	C	C	A
AH2012_12/KU646831/2012/CHN	C	G	G	T	G	T	T	A	T	C	C	T	C	A	C	C	A
85-7-C40/KY486714/2015/CHN	C	G	G	T	G	T	T	A	T	C	C	T	C	A	C	C	A
85-7 mutant	C	G	G	T	G	T	T	A	T	C	C	T	C	A	C	C	A
CH/S/JN547228.1/1986/CHN	C	G	G	T	G	T	T	A	T	C	C	T	C	A	C	C	A
SNJ-P/MK702008/2018/CHN	C	G	G	T	G	T	T	A	T	C	C	T	C	A	C	C	A
OH9097-14/ KP641663/2014/USA	C	G	G	T	G	T	T	A	T	C	C	T	C	A	C	C	A
B5-HB2017/ MF807952/2017/CHN	C	G	G	T	G	T	T	A	T	C	C	T	C	A	C	C	A
LZC/EF185992.1/2007/CHN	C	G	G	T	G	T	T	A	T	C	C	T	C	A	C	C	A
HLJBY/KP403802.1/2015/CHN	C	G	G	T	G	T	T	A	T	C	C	T	C	A	C	C	A
CH_HNLH_2015	C	G	G	T	G	T	T	A	T	C	C	T	C	A	C	C	A
BJ-2011-1/JN825712/2011/CHN	C	G	G	T	G	T	T	A	T	C	C	T	C	A	C	C	A
AJ1102/JX188454/2011/CHN	C	G	G	T	G	T	T	A	T	C	C	T	C	A	C	C	A
CH/F/ND3.2011	C	G	G	T	G	T	T	A	T	C	C	T	C	A	C	C	A
CH/GDZHDM/1401/ KX016034/2014/CHN	C	G	G	T	G	T	T	A	T	C	C	T	C	A	C	C	A

CH/YNKM-8/2013/ KF761675/2013/CHN	C	G	G	T	G	T	T	A	T	C	C	T	C	A	C	C	A
CH/ZJCX-1/2012/KF840537/2012/CHN	C	G	G	T	G	T	T	A	T	C	C	T	C	A	C	C	A
GDS47/MH726382/2016/CHN	C	G	G	T	G	T	T	A	T	C	C	T	C	A	C	C	A
JS-HZ2012/KC210147/2012/CHN	C	G	G	T	G	T	T	A	T	C	C	T	C	A	C	C	A
LNCT2/KT323980/2016/CHN	C	G	G	T	G	T	T	A	T	C	C	T	C	A	C	C	A
PEDV-SX/ KY420075/2015/CHN	C	G	G	T	G	T	T	A	T	C	C	T	C	A	C	C	A
PEDV-WS/ KM609213/2014/CHN	C	G	G	T	G	T	T	A	T	C	C	T	C	A	C	C	A
YN90/ KT021231/2014/CHN	C	G	G	T	G	T	T	A	T	C	C	T	C	A	C	C	A
OK10240-8_2017	C	G	G	T	G	T	T	A	T	C	C	T	C	A	C	C	A

Table S1 Multiple sequence alignment of PEDV strains (continue).

Name	21054	21347	21455	21534	22610	22611	23159	23596	23774	24244	24288	24526	25192	25573	26954	27502
SC-YB73/MT263014/2019/CHN	A	T	C	T	A	C	G	C	C	A	T	G	C	G	A	C
CH-HB2-2018/MK606369.1/2018/CHN	G	C	G	A	G	T	A	T	T	G	C	T	T	A	G	T
CH/JLDH/2016/MF346935/2017/CHN	G	C	G	A	G	T	A	T	T	G	C	T	T	A	G	T
CHN/SH-2016-4/2016/ MG837012/2016/CHN	G	C	G	A	G	T	A	T	T	G	C	T	T	A	G	T
LNSY/KY007140/2015/CHN	G	C	G	A	G	T	A	T	T	G	C	T	T	A	G	T
AH2012_12/KU646831/2012/CHN	G	C	G	A	G	T	A	A	T	G	C	T	T	A	G	T
85-7-C40/KY486714/2015/CHN	G	C	G	A	G	T	A	T	T	G	C	T	T	~	G	T
85-7 mutant	G	C	G	A	G	T	A	T	T	G	C	T	T	A	G	T
CH/S/JN547228.1/1986/CHN	G	C	G	A	G	T	A	T	T	G	C	T	T	A	G	T
SNJ-P/MK702008/2018/CHN	G	C	G	A	G	T	A	T	T	G	C	T	T	A	G	T
OH9097-14/ KP641663/2014/USA	G	C	G	A	G	T	A	T	T	G	C	T	T	A	G	T
B5-HB2017/ MF807952/2017/CHN	G	C	G	A	G	T	A	T	T	G	C	T	T	A	G	T
LZC/EF185992.1/2007/CHN	G	C	G	A	G	T	A	T	T	G	C	T	T	A	G	T
HLJBY/KP403802.1/2015/CHN	G	C	G	A	G	T	A	T	T	G	C	T	T	A	G	T
CH_HNLH_2015	G	C	G	A	G	T	A	T	T	G	C	T	T	A	G	T
BJ-2011-1/JN825712/2011/CHN	G	C	G	A	G	T	A	T	T	G	C	T	T	A	G	T
AJ1102/JX188454/2011/CHN	G	C	G	A	G	T	A	A	T	G	C	T	T	A	G	T
CH/F/ND3.2011	G	C	G	A	G	T	A	T	T	G	C	T	T	A	G	T
CH/GDZHDM/1401/ KX016034/2014/CHN	G	C	G	A	G	T	A	T	T	G	C	T	T	A	G	T

CH/YNKM-8/2013/ KF761675/2013/CHN	G	C	G	A	G	T	A	T	T	G	C	T	T	A	G	T
CH/ZJCX-1/2012/KF840537/2012/CHN	G	C	G	A	G	T	A	T	T	G	C	T	T	A	G	T
GDS47/MH726382/2016/CHN	G	C	T	A	G	T	A	T	T	G	C	T	T	A	G	T
JS-HZ2012/KC210147/2012/CHN	G	C	T	A	G	T	A	T	T	G	C	T	T	A	G	T
LNCT2/KT323980/2016/CHN	G	C	G	A	G	T	A	T	T	G	C	T	T	A	G	T
PEDV-SX/ KY420075/2015/CHN	G	C	G	A	G	T	A	T	T	G	C	T	T	A	G	T
PEDV-WS/ KM609213/2014/CHN	G	C	G	A	G	T	A	T	T	G	C	T	T	A	G	T
YN90/ KT021231/2014/CHN	G	C	G	A	G	T	A	A	T	G	C	T	T	A	G	T
OK10240-8_2017	~	~	G	A	G	T	A	T	T	G	C	T	T	A	G	T
